# Organizing person-centred care in paediatric diabetes: multidisciplinary teams, long-term relationships and adequate documentation

**DOI:** 10.1186/1756-0500-7-72

**Published:** 2014-02-03

**Authors:** Helena Wigert, Ewa Wikström

**Affiliations:** 1 Institute of Health and Care Sciences, The Sahlgrenska Academy, University of Gothenburg, Gothenburg, Sweden; 2 Division of Neonatology, Sahlgrenska University Hospital, Gothenburg, Sweden; 3 Nordic School of Public Health, School of Business, Economics and Law, Gothenburg University, Gothenburg, Sweden

**Keywords:** Health care organization, Long*-*term relationship, Multidisciplinary team, Documentation, Paediatric diabetes, Person-centred care

## Abstract

**Background:**

Type 1 diabetes is one of the most frequent long-term endocrine childhood disorders and the Swedish National Diabetes Register for children states that adolescents (12–18 years) constitute the most vulnerable patient group in terms of metabolic control. The aim of this study was to examine how a multidisciplinary team functions when caring for adolescents with type 1 diabetes.

**Methods:**

Qualitative interviews were performed with 17 health professionals at a Paediatric Diabetes Care Unit in a Swedish university hospital. The interviews were analysed to gain insight into a multidisciplinary care team’s experiences of various organizational processes and circumstances related to the provision of person-centred paediatric diabetes care.

**Results:**

Building long-term relationships with adolescents, the establishment of a multidisciplinary care team and ensuring adequate documentation are vital for the delivery of person-centred care (PCC). Furthermore, a PCC process and/or practice requires more than the mere expression of person-centred values. The contribution of this study is that it highlights the necessity of facilitating and safeguarding the organization of PCC*,* for which three processes are central: 1. Facilitating long-term relationships with adolescents and their families; 2. Facilitating multi-professional teamwork; and 3. Ensuring adequate documentation.

**Conclusion:**

Three processes emerged as important for the functioning of the multidisciplinary team when caring for adolescents with type 1 diabetes: building a long*-*term relationship, integrating knowledge by means of multidisciplinary team work and ensuring adequate documentation*.* This study demonstrates the importance of clearly defining and making use of the specific role of each team member in the paediatric diabetes care unit (PDCU). Team members should receive training in PCC and a PCC approach should form the foundation of all diabetes care. Every adolescent suffering from type 1 diabetes should be offered individual treatment and support according to her/his needs. However, more research is required to determine how a PCC approach can be integrated into adolescent diabetes care, and especially how PCC education programmes for team members should be implemented.

## Background

Type 1 diabetes is one of the most frequent long-term endocrine childhood disorders and the Swedish National Diabetes Register [1] for children states that adolescents (12–18 years) constitute the most vulnerable patient group in terms of metabolic control. Reconciling the demands of diabetes self-care and the developing maturity of adolescents is a major challenge. A common complaint on the part of caregivers of this patient group is that they fail to take sufficiently good care of their health in relation to their disease. In a long-term perspective this could lead to serious medical complications, social problems and a substantial socio-economic burden. Successful prevention of such problems requires that adolescents themselves assume greater responsibility for their self-care, which implies the need for strong social and family support where the school health organization and specialist paediatric care are crucial for creating trust and sustainable alliances. Health care thus plays an important role, but clinical staff members often experience difficulty in overcoming a variety of obstacles connected to the long-term interaction with these patients in view of their personal situation and social context. There are many general guidelines for successful diabetes care [2-4], but there is a lack of information on how to successfully overcome the double hazard of being an adolescent with a life-threatening disease.

Person-centredness (PC) has always been deemed crucial for the delivery of high quality professional care. Although not a new perspective in medical sociology, PC has not been common in the study of medical management and health care organization*,* which mainly focused on management versus professional practice. This study is based on the assumption that PC is essential, but a key empirical question is how to organize, facilitate and safeguard person-centred care (PCC). The essence of PCC comprises patient narratives, extended dialogue as well as the relationship and partnership with the patient [5]. Previous studies [5,6] indicate the importance of focusing on patients’ narratives about or stories of their condition and care needs*,* as well as involving them in the care or treatment. Furthermore, a frequently discussed issue is how to create more integrated care in order to ensure an individualized care organization.

The patient’s personal view has been more or less absent in the organization of the healthcare sector, although recent studies have indicated a transformation in the relationship between the patient and healthcare professionals [7-17]. Obvious changes are related to the increasing consumer orientation and marketization of the healthcare sector. The expectations and visions associated with these changes involve the creation of a care more strongly characterized by PC. A study demonstrated [5] that in order to achieve such care, the organizational process must be guided by bio-psychosocial rather than bio-medical models, as the former focus on the environment and functional ability rather than illness. Attention should also be paid to the patient’s home care needs [17]. Previous studies [18-21] have demonstrated (a) that the aim of PCC is to improve the ethical standard of healthcare in terms of health, autonomy, quality of life and the division of responsibility between patients and care givers; and (b) that increased use of patient narratives and shared decision*-*making may promote this aim.

McCormack et al. [22] stated that, as a concept, PC is becoming more widespread both as an approach to practice and as a guiding principle. Furthermore, they emphasized that despite expressed PC values, the care process remained to a large extent routinized without any effect on the formation of meaningful relationships. In their study they suggested that ‘professional competence’ and ‘knowing self’ are important prerequisites for organizing PCC*.* They stated that earlier studies focused too narrowly on the nurse-patient/family relationship and too little on practitioners’ personal characteristics, ability and competence to form PC relationships. The importance of clinical settings that support PC practice is also underlined. Furthermore, Ekman et al. [5] recommended some routines that could facilitate and safeguard the transition to PCC: 1) initiating the partnership **-** patient narrative, 2) conducting the partnership - shared decision*-*making, and 3) safeguarding the partnership by documenting the narrative.

Previous studies [5,22] have presented some important aspects of healthcare organization in the area of PCC, but there is a lack of empirical research about multidisciplinary care teams’ experiences of various organizational processes and circumstances related to the delivery of PC paediatric diabetes care. Therefore the aim of this study was to examine how a multidisciplinary team functions when caring for adolescents with type 1 diabetes. An additional aim was to contribute to bridging the research gap in the area of medical management and the organization of PCC.

## Methods

### Study design

A qualitative interview method was employed to gain insight into how various organizational processes and circumstances were experienced by the participants. Qualitative methodology using interview narratives explores the phenomena in their natural setting and attempts to make sense of or interpret them in terms of the meanings people attribute to them [23]. Various research strategies for studying processes have unique strengths [24] and in the case of the narrative approach it is accuracy. Pentland [25] stated that the strength of the narrative approach is that it enables us to investigate deep structures of organizational processes that are not directly visible. Thus, the narrative approach is a valuable research tool despite certain weaknesses and limitations.

The interviews contained questions about how the multidisciplinary team focusing on formal and informal interactions, the professionals’ position in relation to the patient and her/his treatment and their perceptions of various aspects of these processes*.* The data were collected through interviews with members of a multidisciplinary team at a Paediatric Diabetes Care Unit (PDCU) in a Swedish university hospital.

### Setting

This study was conducted at a PDCU in a university hospital located in an urban area that is home to a large number of patients of different nationalities. A total of 500 patients aged between 0 and 18 years were registered in the PDCU. The diabetes teams were composed of nurses, nurse assistants, physicians, dieticians, counsellors, psychologists, a medical secretary and play therapists. However, the extent to which these professional categories are involved in regular paediatric diabetes care differs. The patients visit the PDCU during office hours on weekdays every third month for approximately 45 minutes and are examined by the nurses and physicians. A visit to the dieticians, counsellors or psychologists must be requested by the patient or she/he can be referred by the physician.

### Participants

All 17 team members who work with adolescents in the PDCU were invited and agreed to participate in the study. In total 4 nurses, 1 nurse assistant, 4 physicians, 2 counsellors, 1 psychologist, 2 dieticians, 1 medical secretary and 2 play therapists were included. The participants were aged between 38 and 60 years (median 46.5) and their work experience within PDC varied between 0.5 and 24 years (median 6.5).

### Data collection

The interviews were conducted at the PDCU by the first author between June 2011 and May 2012. The interviews were structured into five parts: 1) experiences of working with adolescents with type 1 diabetes; 2) experiences related to the adolescents’ narrative; 3) experiences of encouraging adolescents to take responsibility for their illness and self-care 4) experiences of working with the multidisciplinary team in the PDCU, and 5) experiences of the documentation procedure. The questions were based on the results of pilot interviews with a nurse, assistant nurse, physician and dietician on a paediatric diabetes team in another PDCU with a similar organizational structure to that in the present study. The interview questions were found to be adequate and were therefore used in the study after minor amendments.

The questions were open-ended and the interview started with; “Could you please describe your experience of working with adolescents in the PDCU”? Follow-up questions such as Can you tell me more? and Can you give an example? were posed to clarify statements and confirm that the author had understood the professional correctly. Each interview was digitally recorded and lasted between 33 and 83 minutes. They were then transcribed verbatim and coded from 1–17.

### Data analysis

The analysis, which was conducted by both authors, began by repeated readings of the entire interview text to obtain a sense of the whole./In this phase of the analysis, all transcribed interview texts were carefully studied and coded in accordance with abduction theory methodology [26], which entails a rigid and systematic process of coding and comparing the text, in parallel with the use of theoretical concepts*.* The coding procedure began by labelling the text according to content. These initial codes were then sorted into sub*-*themes and compared with the text. Every passage in the transcripts was labelled by one or more codes that indicated certain recurring topics. The second phase of the analysis concentrated on conceptual development and was broadened to include notes from discussions in the research group. The interviews were then coded at a more conceptual level (focused coding) to describe various processes crucial to the research question. Finally, the sub-themes were compared with the text and three themes emerged (Table 1).

**Table 1:** Outline of themes and sub-themes

**The topics of the interview**	**Themes**	**Sub-themes**
Experiences of working with adolescents suffering from type 1 diabetes	Building long-term relationships	The encounter in the diabetes care unit
		Encountering adolescents with different ethnic backgrounds
Experiences related to the adolescents’ narrative	Building long-term relationships	Encountering the adolescents’ everyday life
Experiences of encouraging adolescents to take responsibility for their disease and self-care	Building long-term relationships	Encountering the adolescents in a relationship
Experiences of working with the multidisciplinary team in the PDCU	Establish a multidisciplinary care team	Working in a team
		Obstacles to the work
Experiences of the documentation procedure	Ensuring adequate documentation	How the meeting was documented
		What was documented
		What was not documented
		Obstacles to documentation

### Ethical aspects

Permission to perform the study was granted by the head of the PDCU. Ethical approval and permission to conduct the study were given by the Regional Research Ethics Committee, registration no. 532–10. All participants were assured that participation was voluntary and that information would be treated confidentially.

## Results

The data analysis revealed that when treating adolescents with type 1 diabetes in the PDCU, the multidisciplinary team used interrelated actions, which are presented as three themes: “Building long-term relationships”, “Establish a multidisciplinary care team” and “Ensuring adequate documentation” (Table 1).

### Building long-term relationships

Encountering adolescents from various backgrounds who suffer from diabetes and helping them to take better care of themselves and their disease, thereby improving their sense of well-being was perceived as challenging. The work was considered important but establishing contact was difficult, as it could take a long time to develop a relationship.

#### The encounter in the diabetes care unit

The team members viewed the visit to the PDCU as an opportunity for the adolescents, although the latter could find it difficult to attend or turn up at the appointed time. It was vital to welcome them, in order to make them feel safe in the encounter.

“*It’s my duty to make them feel that it’s pleasant to come here, I chat with them before they see the doctor, it can loosen them up a bit. Some are on tenterhooks to know what their HbA1c is.*”

The visit to the PDCU was limited to 45 minutes. It was generally regarded as too short, due to, for example, failure to follow up that which had been agreed at the previous encounter. During the visit, focus was on the HbA1c and long-term glucose levels and if these were not good, the adolescent was referred to a nurse to find a solution. The nurses expressed that many adolescents experienced going to the nurse in order to try to work out how to take better care of themselves as a punishment. Adolescents only saw a nurse when there was a problem and they had not managed their diabetes care.

“*Their HbA1c level may be low, and then it’s like a small punishment because they have to see the nurse a few extra times, and if you haven’t met for a long period you have to start building a relationship again.*”

#### ***Encountering adolescents with different ethnic backgrounds***

Several team members described encountering the adolescents as human beings and according to their needs, irrespective of their ethnic background.

“*I treat them the same as any other adolescents, it’s the human being in front of me who I encounter based on her/his needs.*”

The greatest obstacle in the encounter was language; it was difficult to ascertain how much the adolescents and their parents had understood. It was considered vital to have a competent interpreter, due to the risk inherent in the situation.

“*There was no word for hypoglycaemia and I did not realise that the interpreter couldn’t explain it to the family. It only became apparent when the child suddenly had a serious attack.*”

Other difficulties mentioned were the adolescents’ perceived lack of trust in the interpreter’s adherence to the rules of professional confidentiality. When an interpreter was present, the meeting was more time-consuming, which team members found stressful due to their already tight work schedule.

#### ***Encountering the adolescents’ everyday life***

The care of adolescents with diabetes involves addressing their daily lives, including their parents, school and psychosocial situation. If the latter was problematic it had an impact on their ability to take responsibility for their illness and self-care, leading to a low frequency of blood glucose measurement and higher HbA1c levels.

The team members tried to increase the adolescents’ awareness of the disease and inspire a sense of responsibility by motivating them to take care of themselves and praise any progress made. They also focused on the adolescents’ families, as they considered it the parents’ responsibility to ensure that their children experienced well-being despite diabetes. The team members and the family could meet over a period of several years*,* during which time a trusting relationship developed between them.

“*It’s essential to involve the parents, that they understand the seriousness of the situation, sometimes I have to educate parents who suffer from diabetes themselves, although they attend the adult unit.*”

#### ***Encountering the adolescents in a relationship***

In order to encounter adolescents with diabetes, it was necessary to build a relationship with them by taking an interest in their life situation, which required time.

“*A relationship cannot be built by seeing each other a few times for 5 minutes.*”

The staff members endeavoured to create trust in the relationship as a means of making the adolescents dares to open up and narrate about unsatisfactory aspects of their lives.

“*I had to file a report with the social services when he told me that he used hash, but he continued to come to me. When I asked him why he came, he answered, because you care.*”

### The establishment of a multidisciplinary care team

All professional groups that participated in this study were represented on the diabetes unit team, which met once per week. They worked in different parts of the unit and therefore the team conferences were regarded as a meeting place.

#### ***Working in a team***

The work was based on a team model… *“How else could we work?”.* A strength was that the team members represented different professional groups. The advantage of this mode of working was that the team members helped each other. They experienced receiving emotional as well as practical support by discussing with fellow members how they could best help the adolescents to experience greater well-being. They referred their patients to the professional group that they considered most suitable.

“*When they get stuck, are unable to reach a child, the play therapists are called in to wave their magic wand, which is not easy.*”

Some members had a stronger sense than others of belonging to the team, while certain professional groups did not feel that their participation was self-evident.

“*You feel a bit like an outsider when you don’t have a medical education.*”

The importance and function of the various professional groups in the care of adolescents with diabetes were reflected in the team.

“*We have two large professional groups, doctors and nurses, and they have the greatest say, it’s a medical hospital and this is reflected in the team.*”

Lack of continuity was experienced as an organizational problem. Some professional groups acted more like consultants than team members and cases could be discussed that did not concern them.

“*Much of the time is spent talking about cases that don’t concern me as a school welfare officer, it sometimes feels like a meeting that you could just as well miss.*”

There was a wish to enhance the team spirit, and for this purpose a suggestion was made that teamwork tools and models be used. The staff members were of the opinion that by working towards a common goal they would be better able to meet the adolescents’ needs.

#### ***Obstacles to the work***

The team members experienced that the main obstacle in their work with the adolescents was the stressful work situation with a high tempo and difficulty deviating from the planned daily schedule. Reduced financial resources led to competition between the hospital’s activities and units.

“*It’s always a struggle to obtain resources, the diabetes unit wants to employ more dieticians, but the answer is always no, yet we could make a difference.*”

A lack of clarity regarding one’s remit was also reported *…you don’t always know what is expected of you*, which meant that the duties were sometimes not experienced as relevant. Communication between staff and management suffered due to the high pace of work. The professional categories that acted as consultants only saw the adolescents after they had received the diagnosis of diabetes or were referred by one of the doctors. They continued to meet the adolescents until their ‘problems’ were considered solved and there was no time for follow up… “*I don’t know how much of the advice I have given works in the home, instead I have to trust the doctors to follow it up*”*.* It was also quite common for a new referral containing the identical question regarding the same adolescent to appear after about a year, *…*“*you have a feeling that it will arise again and it takes three months until the child is readmitted or a referral arrives from the doctor reporting that it doesn’t work*”*.*

### Ensuring adequate documentation

All staff categories documented the meeting with the adolescent in her/his medical record with the exception of the secretary who just documented physicians’ that which the physicians dictated and play therapists, who informed the nurse that something needed to be documented.

#### ***How the meeting was documented***

The most frequent method of documentation was catchwords, which was mainly employed by team members to remind themselves of what had been said and done prior to their next meeting with the adolescent. It was important to be able to*… “remind the patient that she told me the last time that she crochets small things, it creates trust when you remember such details”.* The professionals wrote brief entries in the medical record when they were short of time; it was usual to summarise the meeting in a few words.

“*I’m the only secretary, have a lot to do, I plead with them not to make the quotations too long.*”

Some of the professionals documented the encounter in consultation with the adolescents when important agreements had been made concerning their diabetes care or when sensitive issues had been discussed.

#### ***What was documented***

Test results were documented as well as how the adolescents experienced living with the disease. In addition, their life situation was documented, as it was considered important for the care and of value to other staff members.

“*I document that which is important for understanding this human being.*”

They considered it their duty to document situations that could lead to a report to the social authorities when the adolescents were exposed to harm and also wanted to demonstrate that the situation had been noted… “*it’s important to document parental abuse, as the adolescent might meet a colleague at a subsequent visit*”*.*

#### ***What was not documented***

The team members found it difficult to judge what should and what should not be documented. They sometimes chose not to document when they perceived that it could be negative for the adolescents. However, in some cases the patient did not wish what she/he had narrated to be documented.

“*A serious incident was revealed, which the patient didn’t want me to document. She trusted me and I don’t think she would ever have trusted anyone in the health service again had I let her down.*”

Heart-to-heart conversations between the adolescents and team members were often not documented.

#### ***Obstacles to documentation***

Some team members regarded it as an obstacle when colleagues chose not to document details about the adolescent’s situation or their notes were fragmented. They lacked documentation of*… “the small nuances, detailed explanations, which opened the lock, concerning the routine rather than the individual level”.* A record common to all professional groups, where they could document and read notes regarding the adolescent’s care, would be desirable.

## Discussion

The study reveals three important processes in multidisciplinary team work when caring for adolescents with type 1 diabetes: building long*-*term relationships, integrating knowledge by means of multidisciplinary team work and ensuring adequate documentation. The first requires extensive communication between the professionals and the adolescent based on trust, mutual responsibilities and ethical considerations. The second demands communication between the professional groups, while the third necessitates a balance between what kind of documentation could become an obstacle for the adolescent in the future and documentation that safeguards the functioning of the multidisciplinary team.

Positive benefits are expected from multidisciplinary team work, especially in healthcare. In the last decade calls have been made for healthcare organizations to integrate their various areas of expertise and different skills in order to provide PCC that is more strongly focused on patients as a whole in their unique medical situations. In healthcare, multidisciplinary teams may be the organizational solution to providing improved PCC. However, it is not clear what is meant by PCC or what distinguishes it from standard care planning models and medical decision*-*making ideals. PCC can be understood as a caring relationship between the carer, patient and her/his family [5,22], which is supported by the present study where professionals’ relationship with the adolescents and her/his family was central. A caring relationship requires encountering the patient based on each family’s everyday life [27-29] and is developed when the patient her/himself plays an active role [30], which is the case in PCC. The core of PCC is a meaningful relationship [22] often associated with shared decision*-*making [18] and it is thus necessary to create such a relationship with the patient [5]. The team members described their encounters with the adolescents in the PDCU as being built on the relationship between them. However, creating a relationship requires time and continuity, which were often lacking, as team members were pressed for time and did not always meet ‘their’ patients. They were aware of the importance of establishing a relationship, the fact that adolescence is a special phase of life and that communication and language are essential, although the latter aspect was not mentioned in the guidelines and goals that formed the basis of their work. This conclusion accords with earlier research emphasizing the importance of a partnership based on the patient narrative [5,6,15-22]. Furthermore, our study also highlights the observation by McCormack et al. [22] that expressing PC values does not guarantee a PCC process or practice.

From a medical management perspective, PCC is about treating people with dignity and respect, empowering them and giving them a choice as well as involving and making them co-actors in the care [5]. It can be argued that the central figure and key member of the team is the patient her/himself. However, none of the informants in this study mentioned this aspect. Perhaps the question of how to enhance the encounter between adolescents and team members so that both experience it as an improvement should be raised in the team. Other questions that need to be highlighted are what improvement potential exists in the team and why some professional categories only encounter the adolescents due to referrals from colleagues when problems occur*.* Is it possible to improve the organisation of the team? Can the work situation be improved?

The informants in the present study held that different professional groups should work in teams in order to provide optimal conditions for high quality care, which also accords with other studies [31,32]. However, team work can mean and involve different things to team members. In order to benefit the patient, team work must function well and team members have to experience themselves as participants. The organisation of which the team is a part has an obligation to ensure that this is the case [32].

It is essential that the members experience themselves as a team, share the same goal, values and holistic view of how situations should be solved [32]. Several of the participants stated that they are a team but at the same time work individually and have no common documentation, goal or organisation. They do not utilise the dynamics inherent in a team but perceive that their work is characterised by a tradition that it is hierarchical in character. There is a clear division of roles and leadership in the team, which is in line with Kvarnström [31]. Although the professional role is strengthened by team work, team dynamics can be perceived as threatening the status of the respective professional category and the hierarchical character of the organisation when different professional groups cooperate. Working in a team can be experienced as frustrating when a sense of not belonging is present [31]. To make fruitful teamwork possible, it is important to leave one’s own professional affiliation and group behind and instead identify oneself as ‘we’ , part of the team [32].

In terms of documentation, the results demonstrate that no PC team documentation existed in the studied PDCU and there appears to be a lack of guidelines for how and what to document. The above pattern reveals that the multidisciplinary team coordinates the care as opposed to focusing on interprofessional collaboration to integrate the treatment. This conclusion is supported by previous findings on multidisciplinary teamwork in healthcare*,* where the positive outcome mainly relates to coordination [33,34] and, to a lesser extent, to knowledge sharing [35-37].

PCC highlights the importance of the care being based on the patient’s experiences of her/his disease and life situation [22], which was also underlined by the participants in the present study. In order to apply PCC, the carer has to take an interest in the patient as a person and utilise the encounter to gain knowledge of her/his everyday life. One way of implementing PCC is by encountering the patient in a caring relationship*.*

This study has strengths as well as limitations. The research was designed to examine the professionals’ perspectives and experiences in the studied settings; i.e. the data permit access to the professional groups but not to the patient group. Another limitation is that the study was conducted with only one team in one PDCU and that there were no observations. However, the PDCU had a large number of patients with various social and ethnic backgrounds*,* which can be seen as strength.

## Conclusion

The multidisciplinary care team for treating adolescents with type 1 diabetes is characterized by three important processes: Building long-term relationships with adolescents, the establishment of a multidisciplinary care team and ensuring adequate documentation. Our study supports the results of earlier research that PCC values alone are insufficient to create a PCC process and organize PCC [5,22]. Nevertheless, in the present study the multidisciplinary team facilitated and safeguarded the organization of PCC. Furthermore, the main challenge is to ensure well-functioning multi-disciplinary collaboration and avoid organizing the care according to traditional healthcare paradigms, in which administrative and professional boundaries create obstacles for adolescents with chronic multi-faceted illnesses.

This study demonstrates the importance of clearly defining and employing the specific role of each team member in the PDCU. Team members should be educated in PCC and a PCC approach should constitute the foundation of all diabetes care. Adolescents with type 1 diabetes should be offered individual treatment and support based on their needs. However, more research is needed on how a PCC approach can be integrated into adolescent diabetes care, and especially how PCC education programmes for team members should be implemented.

## Abbreviations

PCC: Person-centred care; PDCU: Paediatric diabetes care unit.

## Competing interests

The authors report that they have no competing interests.

## Authors’ contributions

HW and EW contributed to the conception and design of the study. HW performed the data collection. HW and EW analysed and interpreted the data and wrote the final manuscript. Both authors read and approved the final manuscript.

## Authors’ information

Helena Wigert is Senior Lecturer at the Institute of Health and Care Sciences, The Sahlgrenska Academy at the University of Gothenburg and the Division of Neonatology, Sahlgrenska University Hospital, Gothenburg, Sweden.

Ewa Wikström is Professor at the Nordic School of Public Health and The School of Business, Economics and Law at Gothenburg University.
